# Predicting 1-year mortality in older cancer patients: performance of G8, SPPB, and IF-VIG in the PROFIT Study

**DOI:** 10.1093/gerona/glag099

**Published:** 2026-04-16

**Authors:** Maria Cristina Ferrara, Fabricio Zambom-Ferraresi, Aida Ribera, Idoia Morilla-Ruiz, Sofia Espinoza-Tofalos, Carmina Castellano-Tejedor, Josep Majó-Llopart, Oriol Planesas-Pérez, Eva Heras, Francisco José Tarazona-Santabalbina, Hugo Badani, Nicolàs Martínez-Velilla, Marco Inzitari

**Affiliations:** School of Medicine and Surgery, University of Milano-Bicocca, Monza, Italy; IdiSNA, Navarrabiomed, Hospital Universitario de Navarra (HUN), Department of Geriatric Medicine, Universidad Publica de Navarra (UPNA), Pamplona, Spain; CIBER of Frailty and Healthy Aging (CIBERFES), Instituto de Salud Carlos III, Madrid, Spain; REFiT research group, Parc Sanitari Pere Virgili and Vall d’Hebron Institute of Research, Barcelona, Spain; CIBeR de Epidemiología y Salud Pública (CIBERESP), Madrid, Spain; IdiSNA, Navarrabiomed, Hospital Universitario de Navarra (HUN), Department of Geriatric Medicine, Universidad Publica de Navarra (UPNA), Pamplona, Spain; CIBER of Frailty and Healthy Aging (CIBERFES), Instituto de Salud Carlos III, Madrid, Spain; Division of Geriatric and Intensive Care Medicine, Careggi Hospital, University of Florence, Florence, Italy; REFiT research group, Parc Sanitari Pere Virgili and Vall d’Hebron Institute of Research, Barcelona, Spain; Department of Psychology and Education Sciences, Universitat Oberta de Catalunya (UOC), Barcelona, Spain; Institut Català d’Oncologia, Girona, Sant Ponç, Avinguda de França 0, Girona, Spain; REFiT research group, Parc Sanitari Pere Virgili and Vall d’Hebron Institute of Research, Barcelona, Spain; Servei Envelliment i Salut, Servei Andorrà d’Assistència Sanitària, Carrer Dels Escalls, S/N, Escaldes, Andorra; Geriatric Service, Hospital Universitario de la Ribera, Valencia, Spain; REFiT research group, Parc Sanitari Pere Virgili and Vall d’Hebron Institute of Research, Barcelona, Spain; Geriatric Service, Parc Sanitari Pere Virgili and Vall d’Hebron Institute of Research, Carrer d‘Esteve Terradas, Barcelona, Spain; IdiSNA, Navarrabiomed, Hospital Universitario de Navarra (HUN), Department of Geriatric Medicine, Universidad Publica de Navarra (UPNA), Pamplona, Spain; CIBER of Frailty and Healthy Aging (CIBERFES), Instituto de Salud Carlos III, Madrid, Spain; REFiT research group, Parc Sanitari Pere Virgili and Vall d’Hebron Institute of Research, Barcelona, Spain; Department of Psychology and Education Sciences, Universitat Oberta de Catalunya (UOC), Barcelona, Spain

**Keywords:** Frailty, Geriatric assessment, Physical performance, Cancer

## Abstract

**Background:**

Frailty is a prognostic determinant in older patients, yet the most effective tools to predict survival remain unclear. This study aimed to assess the predictive performance of different frailty assessment tools for 1-year mortality in the oncogeriatric population.

**Methods:**

A multicenter cohort study (PROFIT Study) involved patients aged ≥65 with cancer, evaluated in oncology clinics and post-acute oncogeriatric units. Frailty was measured using the Geriatric 8 questionnaire (G8), Short Physical Performance Battery (SPPB), and the Frailty Index Indice Frágil—Valoración Integral Geriátrica (IF-VIG). One-year mortality was monitored. Predictive ability was analyzed using receiver operating characteristic curves with optimized cut-offs, and covariate-adjusted Cox regression models were used to evaluate the association between frailty and mortality.

**Results:**

Among 229 patients (mean age 75.1 ± 6.4 years; 68.6% male; cancer type: 47.2% lung cancer, 17.9% colorectal, 25.3% other gastrointestinal, 9.6% prostate; tumoral stage IV: 85.2%), 146 (63.7%) died within 1 year. All tools showed predictive value, with IF-VIG demonstrating the highest sensitivity and SPPB the highest specificity. Optimized cut-offs improved performance compared to standard thresholds (G8: 12.5 vs 14; SPPB: 8 vs 9; IF-VIG: 0.16 vs 0.25). Adjusted Cox models confirmed significant associations with 1-year mortality: hazard ratio [HR] 1.97 (95% CI 1.30–2.99) for G8, 2.35 (95% CI 1.52–3.64) for SPPB, and 2.42 (95% CI 1.50–3.90) for IF-VIG.

**Conclusions:**

All frailty tools were significantly associated with 1-year mortality. SPPB and IF-VIG outperformed G8 in prognostic accuracy, highlighting their potential utility in clinical decision-making for older patients with cancer.

## Graphical Abstract

**Graphical Abstract:**
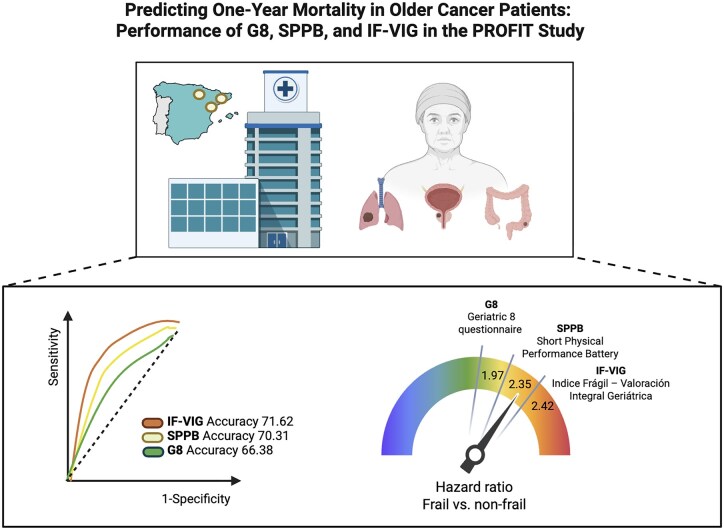

## Introduction

Frailty is a dynamic state of reduced physiological reserve that increases older adults’ vulnerability to adverse health outcomes,^1–3^ particularly in cancer patients who face multiple disease- and treatment-related stressors.^4^^,^^5^ Frail cancer patients may deteriorate quickly, reaching a stage where disease-specific treatments provide limited or no meaningful benefit. Frailty assessment can therefore help avoid interventions that would substantially impair quality of life or, conversely, guide the use of cancer-specific treatments when the patient is not frail, regardless of chronological age.^6^

A recent systematic review of 102 studies reported that the likelihood of receiving anticancer treatment decreases with increasing age, highlighting the urgent need for more evidence to better tailor treatment decisions in older patients.^7^ Conversely, healthcare delivered in the last year of life for advanced cancer is often costly and may provide limited value for some patients, particularly the frailest.^8^ In this context, accurate prognostication over a clinically meaningful horizon—usually operationalized as 12-month survival—can guide care planning and shared, informed decision-making.^9^ Accordingly, commonly adopted identification approaches explicitly consider the likelihood of death within the next 12 months (eg, the “Surprise Question”^10^, NECPAL^11^), supporting timely goals-of-care discussions. By framing prognosis within an understandable timeframe, patients and healthcare professionals can better weigh expected survival against treatment burden and patient-centered outcomes such as quality of life and function.^12^ This information can directly inform discussions on whether to initiate, continue, de-escalate treatments, or prioritize supportive/palliative approaches. Therefore, 1-year mortality provides an actionable horizon to benchmark frailty screening instruments in older adults with cancer.

Although frailty assessment is central to geriatric care, the most effective tools to predict survival in older cancer patients remain unclear.^13^^,^^14^ The Geriatric 8 questionnaire (G8) is designed to identify patients who may benefit from a Comprehensive Geriatric Assessment (CGA),^15^ yet its prognostic utility is not fully established. CGA is the gold standard for multi-domain frailty assessment,^16–18^ and the Indice Frágil—Valoración Integral Geriátrica (IF-VIG) derives a frailty index from a standardized CGA.^19^ However, CGA-based tools can be time-consuming and resource-intensive,^20^ which has prompted interest in screening methods that are both effective and feasible, even in settings without dedicated geriatric specialists.^21^^,^^22^ The Short Physical Performance Battery (SPPB) evaluates physical function^23^ and has been suggested as a predictor of survival.^24–26^ However, evidence on the ability of these tools to predict 1-year mortality in older cancer patients remains limited.

Therefore, this study aimed to compare the predictive performance of G8,^15^ SPPB,^23^ and IF-VIG^19^ and to evaluate the association between frailty, measured with the 3 tools, with 1-year mortality in this population.

## Method

### Study population

This multicenter cohort study was conducted within the PROFIT Study (PeRsonalizing the approach to the Oncologic Frail Individual through Tailored assessment and intervention—Work Package 1), to address current gaps in evidence-based oncogeriatric care. Data were collected from 3 different Spanish centers: the post-acute oncogeriatric unit at Parc Sanitari Pere Virgili in Barcelona (which provides care for complications following oncologic treatments and offers geriatric rehabilitation for patients with advanced cancer), and the outpatient oncologic clinics of Hospital Universitario de Navarra (HUN) in Pamplona and Institut Català d‘Oncologia (ICO) in Girona.

The inclusion criteria were (1) age ≥65 years; (2) diagnosis of lung, prostate, or gastrointestinal solid tumors, with T3-T4 extension, involving lymph nodes (*N* ≥ 1) either metastatic or not (M0-1), with or without ongoing oncologic treatment; (3) evidence of mild functional impact, based on Barthel Index ≥ 50^27^; (4) life expectancy ≥ 3 months; and (5) willingness to provide informed consent to participate. Patients with moderate-to-severe cognitive impairment (Global Deterioration Scale ≥ 5^28^) were excluded. All patients admitted to the participating centers satisfying the eligibility criteria received detailed verbal and written explanations of the characteristics and objectives of the study, and those who agreed were included in the study after signing a written informed consent form. In this study, we additionally excluded patients with missing baseline frailty evaluation data (*n* = 19).

The study was approved by the Research Ethics Committees of the Universitat Autònoma de Barcelona (CEEAH 4946, 31/01/2020) and HUN (PI_2019/117, 17/12/2019), and it was notified to the local committee of ICO. Data were recorded, after anonymization, in a RedCap database (developed by Vanderbilt University, Nashville, TN) for multi-centric research, freely licensed for the REFiT Barcelona group and hosted in a dedicated server with respect to the national regulations regarding data storage and protection. The data analysis was conducted centrally.

### Frailty assessment tools

Baseline assessment was conducted by trained healthcare professionals. Frailty was measured using the following tools: G8, an 8-item questionnaire assessing age, nutritional status, basic mobility, neuropsychological problems, number of drugs, and health perception; the final score is usually considered abnormal if ≤ 14 points^15^; SPPB, comprising 3 timed tasks (chair stand, balance, and walking speed tests) to assess physical performance, extensively described as a frailty indicator if ≤ 9 points^23^; IF-VIG, a frailty index based on CGA, with ≥ 0.25 points usually indicating frailty^19^ according to the cumulative deficit theory validated in aging research.^29^

### Other variables

Sociodemographic characteristics (age, sex, education, civil status, and living situation), as well as oncological variables (primary site of cancer, tumoral stage, treatments, ie, surgery, chemotherapy, radiotherapy), comorbidity burden (Cumulative Illness Rating Scale for Geriatrics [CIRS-G]),^30^ functional status (Barthel Index^27^), Eastern Cooperative Oncology Group [ECOG] performance status scale^31^), cognition (Mini-Mental State Examination),^32^ and quality of life perception (EuroQoL-5D-3L [EQ-5D-3L])^33^ were collected.

### Mortality outcome

Patient 1-year survival was monitored through follow-up visits conducted at 3, 6, and 12 months.

### Statistical analysis

The baseline characteristics of the sample were analyzed using central tendency and dispersion measures. Means and *SD* were used for normally distributed data, while medians and interquartile range (IQR) were applied to non-normally distributed data. Categorical variables were summarized as counts and percentages. Comparisons of baseline characteristics between living and dead patients were conducted using *t*-tests or Mann–Whitney *U* tests for continuous variables, depending on their distribution, and chi-square or Fisher’s exact tests for categorical variables. Receiver operating characteristic curves were constructed for each frailty tool by plotting sensitivity against 1-specificity across a range of thresholds to evaluate their ability to discriminate between patients who survived versus those who did not within 1 year. The area under the curve (AUC) was calculated as a summary measure of each tool’s discriminative power, with the Youden Index pinpointing the optimal cut-off point by identifying the threshold that maximizes the combined sensitivity and specificity.^34^^,^^35^ Bootstrap simulation was used to compare AUCs. Calibration was assessed by plotting the observed versus expected frequencies of mortality at 12 months in each risk decile with a 95% CI.

Kaplan–Meier curves were generated to illustrate mortality rates over a year using the identified optimized cut-off values for each frailty tool. Finally, Cox proportional hazards models were used, after confirming no violation of the proportional hazards assumption, to explore the association between frailty and 1-year mortality risk. Separate Cox models were constructed for each frailty tool with optimized cut-off values, generating hazard ratios (HR) and 95% CI for 1-year mortality risk. Each model was adjusted for age, sex, education (middle school or above), comorbidity burden (CIRS-G), functional status (Barthel Index), and primary cancer site (colorectal, prostate, or other gastrointestinal vs lung as reference). Covariates were selected based on clinical relevance and prior literature.^36^^,^^37^ Sensitivity analyses additionally adjusted for tumoral stage, and repeated the models replacing the Barthel Index with ECOG performance status. Analyses were conducted using R 4.3.2 (Copyright © 2023 The R Foundation for Statistical Computing, Vienna, Austria) software version.

## Results

**Figure 1:**
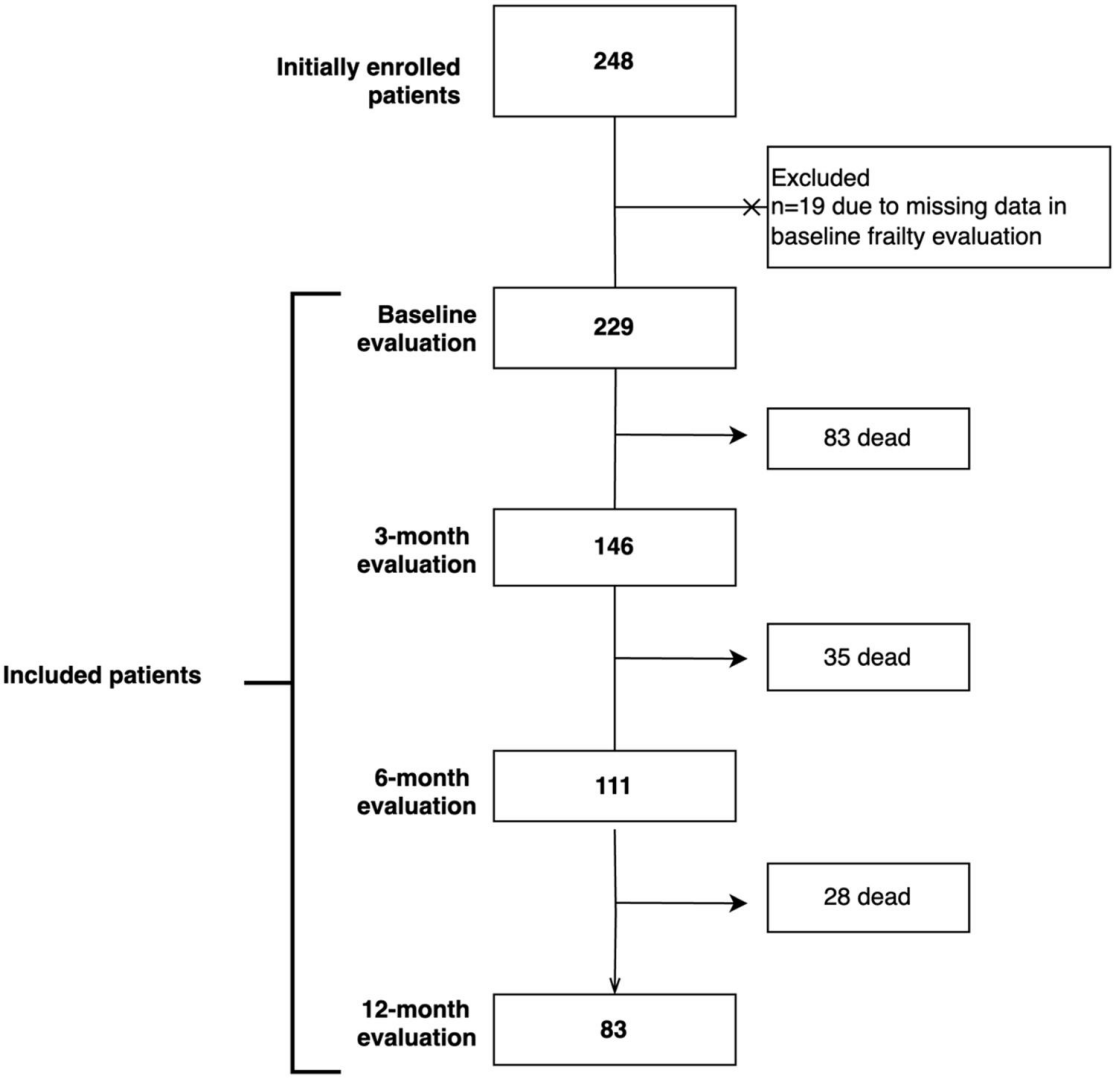
Flowchart of the study.

A total of 229 patients were included (mean age 75.1 ± 6.4 years; 68.6% male), of whom 146 (63.7%) died over 1 year of follow-up (Figure 1). Approximately two-thirds of participants were recruited from outpatient clinics, and one-third from the post-acute oncogeriatric unit. Most patients lived with a partner, had a low educational level, and exhibited a moderate comorbidity burden (Table 1). Lung and gastrointestinal cancers were the most prevalent primary sites, accounting for 90% of cases, and 85.2% of patients presented with stage IV disease. Chemotherapy was administered to the majority, while less than half received radiotherapy. Baseline Barthel Index was 90 (IQR 70-100), and cognitive impairment was uncommon (absence of cognitive impairment: 96.5%). Health-related quality of life (EQ-5D-3L index) was 0.7 (IQR 0.5-0.8). Frailty measures showed a G8 score of 11.5 (IQR 9-14.5), an SPPB score of 7 (IQR 3-10), and an IF-VIG frailty index of 0.20 (IQR 0.10-0.40).

**Table 1:** Patient baseline characteristics (overall and according to 1-year mortality).

Variables^a^	Overall	Alive	Dead	*p*-Value
	(*n* = 229)	(*n* = 83)	(*n* = 146)	
**Age, mean (*SD*)**	75.1 (6.4)	74.5 (5.7)	75.4 (6.7)	.427
**Male sex, *n* (%)**	157 (68.6)	48 (57.8)	109 (74.7)	.013
**Clinical setting, *n* (%)**				.051
Post-acute oncogeriatric care	69 (30.1)	18 (21.7)	51 (34.9)	
Outpatient oncologic clinics	160 (69.9)	65 (78.3)	95 (65.1)	
**Married/in couple, *n* (%)**	149 (65.4)	58 (69.9)	91 (62.8)	.346
**Living alone, *n* (%)**	63 (27.5)	25 (30.1)	38 (26)	.608
**Education (illiterate or primary), *n* (%)**	134 (58.5)	48 (57.8)	86 (60.1)	.937
**Barthel Index, median (IQR)**	90 (70-100)	100 (85-100)	85 (65-95)	<.001
**CIRS-G, median (IQR)**	5 (3-8)	3.5 (2-7)	6 (4-9)	<.001
**Absence of cognitive impairment**	221 (96.5)	81 (97.6)	140 (95.9)	.714
**Primary cancer site, *n* (%)**				.212
Lung	108 (47.2)	40 (48.2)	68 (46.6)	
Colorectal	41 (17.9)	14 (16.9)	27 (18.5)	
Prostate	22 (9.6)	12 (14.5)	10 (6.8)	
Other gastrointestinal	58 (25.3)	17 (20.5)	41 (28.1)	
**Tumoral stage IV, *n* (%)**	190 (85.2)	62 (74.7)	128 (91.4)	.001
**ECOG, *n* (%)**				.042
0-2	187 (81.7)	74 (89.2)	113 (77.4)	
3-4	42 (18.3)	9 (10.8)	33 (22.6)	
**Surgical treatment, *n* (%)**	57 (25.4)	27 (33.3)	30 (21)	.06
**Chemotherapy, *n* (%)**	186 (81.9)	68 (81.9)	118 (81.9)	1
**Radiotherapy, *n* (%)**	92 (40.7)	39 (47)	53 (37.1)	.186
**EQ-5D-3L index score, median (IQR)**	0.7 (0.5-0.8)	0.8 (0.6-1)	0.6 (0.4-0.7)	<.001
**G8 median (IQR)**	11.5 (9-14.5)	13 (10-15)	10.8 (8-13)	<.001
**SPPB, median (IQR)**	7 (3-10)	10 (6.5-12)	6 (3-9)	<.001
**IF-VIG median (IQR)**	0.2 (0.1-0.4)	0.1 (0.1-0.2)	0.2 (0.2-0.4)	<.001

Abbreviations: CIRS-G, Cumulative Illness Rating Scale for Geriatrics; ECOG, Eastern Cooperative Oncology Group performance status scale; EQ-5D-3L, EuroQoL-5D-3L; G8, Geriatric 8 questionnaire; IF-VIG, Indice frágil—Valoración Integral Geriátrica; IQR, interquartile range; SPPB, Short Physical Performance Battery.

a Total *n* for variables with missing data: married/in couple (*n* = 228), surgical treatment (*n* = 224), chemotherapy (*n* = 227), radiotherapy (*n* = 226), tumoral stage (*n* = 223), and EQ-5D-3L index score (*n* = 223).

Patients who died were more often male and showed poorer functional status, higher comorbidity burden, more advanced tumor stage, lower baseline quality of life, and higher frailty scores. No significant differences were observed in treatment approaches between survivors and non-survivors.

**Figure 2:**
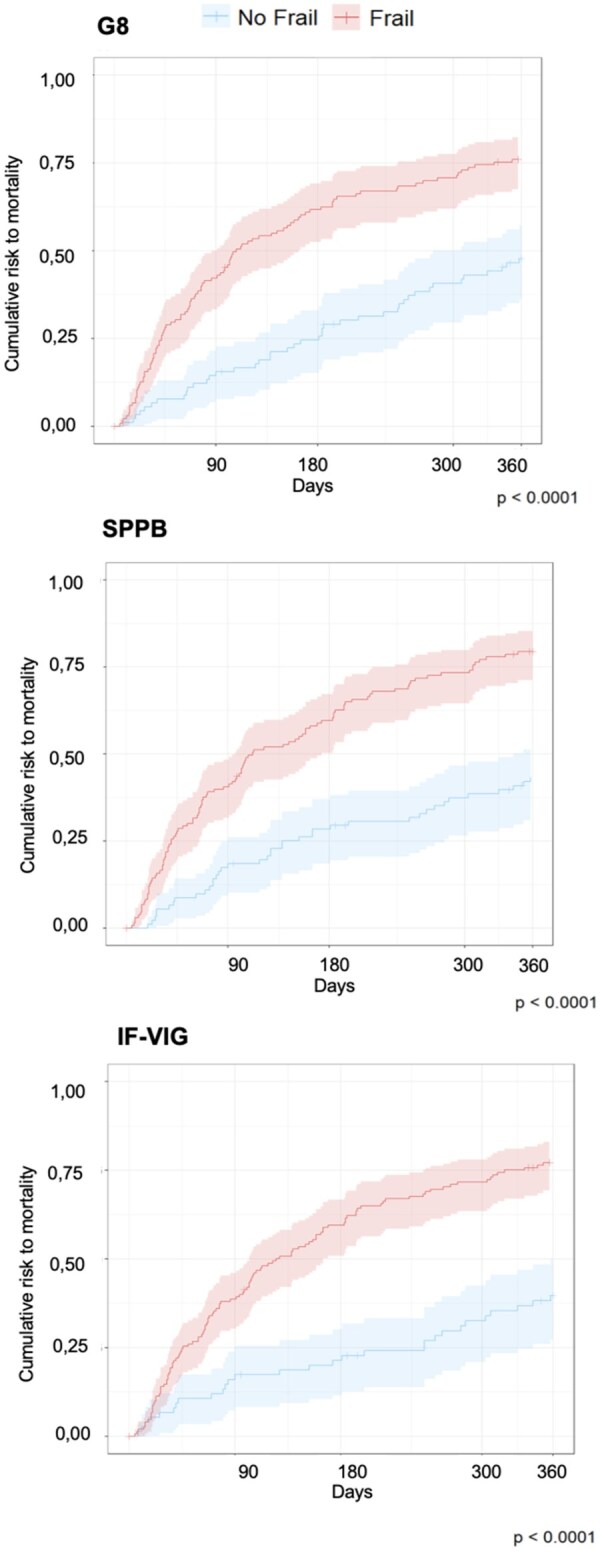
Optimal cut-off values for G8, SPPB, IF-VIG in predicting 1-year mortality (determined by ROC curve analysis and Youden Index).

All 3 frailty tools significantly predicted 1-year mortality. The AUC values were 0.669 (95% CI 0.595-0.743) for G8, 0.727 (95% CI 0.656-0.798) for SPPB, and 0.732 (95% CI 0.661-0.804) for IF-VIG (Figure 2). While both SPPB and IF-VIG outperformed G8 in discrimination, statistical significance was reached only for IF-VIG (*p*-values for AUCs difference *p* = .032 for IF-VIG vs G8; *p* = .077 for SPPB vs G8). IF-VIG showed the highest sensitivity, whereas SPPB had the highest specificity and greater sensitivity than G8. Optimized cut-offs for frailty were lower than standard thresholds (G8: ≤12.5 vs ≤14; SPPB: ≤8 vs ≤9; IF-VIG: ≥0.16 vs ≥0.25). Calibration plots are provided in the Supplementary Materials (Figures S1–S3). Table S1 shows the reclassification of frailty status according to the original and optimized cut-offs for each frailty tool, whereas the Venn diagram (Figure S4) illustrates the partial overlap among frailty classifications based on G8, SPPB, and IF-VIG after cut-off optimization, with 100 patients identified as frail by all 3 instruments and a minority identified as frail by 1 or 2 tools.

**Figure 3:**
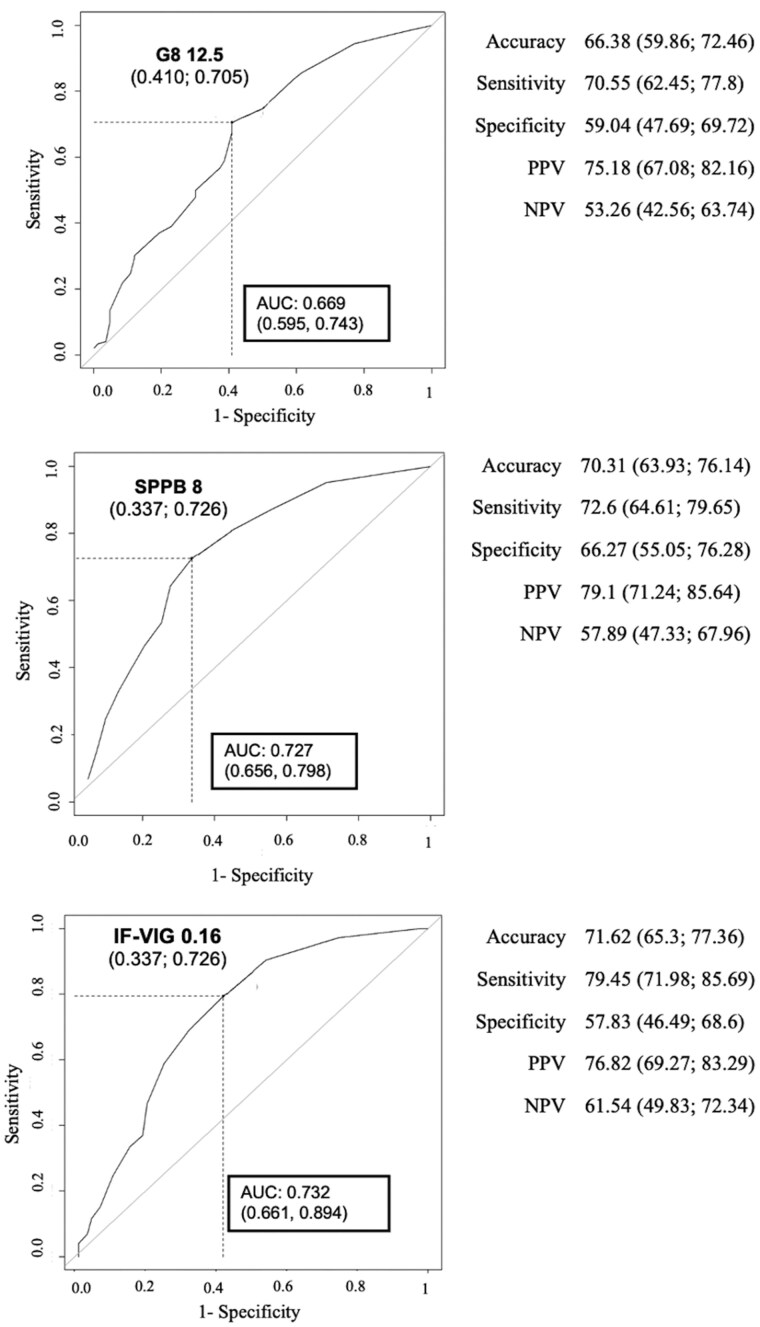
Mortality at 1 year estimated by Kaplan–Meier method. Abbreviations - AUC: Area Under the Curve; G8: Geriatric 8 questionnaire; SPPB, Short Physical Performance Battery; IF-VIG Indice frágil - Valoración Integral Geriátrica.

**Figure 4:**
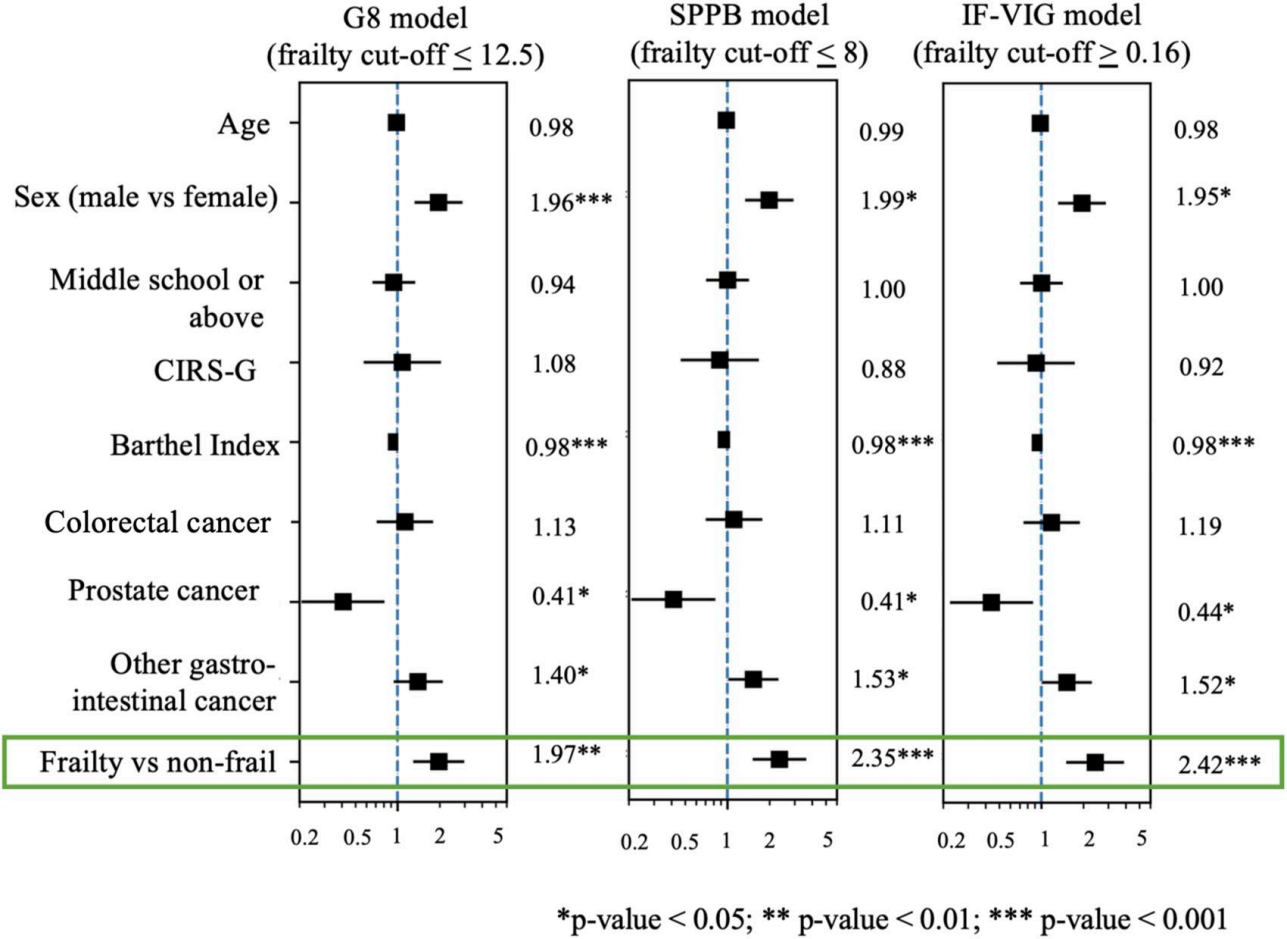
Association between frailty and risk of 1-year mortality (adjusted for age, sex, education, comorbidity burden, functional status, and primary cancer site). Note: Lung cancer is used as a reference for any cancer site. Abbreviations - CIRS-G: Cumulative Illness Rating Scale for Geriatrics; G8: Geriatric 8 questionnaire; SPPB, Short Physical Performance Battery; IF-VIG Indice frágil - Valoración Integral Geriátrica.

Finally, Kaplan–Meier survival curves illustrated mortality differences according to optimized frailty cut-offs (Figure 3). In multivariable Cox proportional hazards models, frailty remained significantly associated with 1-year mortality after adjustment for age, sex, education, CIRS-G, Barthel Index, and primary cancer site (Figure 4). The strongest associations were observed for IF-VIG (HR 2.42, 95% CI 1.50-3.90; *p* < .001) and SPPB (HR 2.35, 95% CI 1.52-3.64; *p* < .001), compared with G8 (HR 1.97, 95% CI 1.30-2.99; *p* < .01). Similar results were obtained after additional adjustment for tumoral stage (Figure S5), as well as after substituting Barthel Index with ECOG performance status as a covariate (data not shown).

## Discussion

In this multicenter cohort of older cancer patients, G8, SPPB, and IF-VIG all demonstrated significant prognostic value for 1-year mortality, with SPPB and IF-VIG showing the highest predictive accuracy and strongest associations with mortality. These findings reinforce the importance of frailty assessment in oncogeriatric care.

The high 1-year mortality rate observed aligns with existing evidence, emphasizing the need for reliable prognostic tools.^38^^,^^39^ Non-survivors were more likely to have a higher comorbidity burden—reflecting the complex interplay between multiple chronic conditions, cancer, and mortality in older adults^40^—as well as poorer baseline functional status, and lower quality of life, consistent with recent evidence showing that frail older cancer patients frequently experience substantially reduced quality of life.^41^

Our results expand on previous studies confirming frailty as a robust predictor of mortality.^4^^,^^5^ A systematic review has shown that G8 was associated with mortality in 15 of 24 included studies,^42^ while objective measures of physical function, such as SPPB and walking speed, independently predict survival.^25^ Prior research on elective oncological surgery similarly highlighted the predictive value of CGA-derived frailty indices, with AUCs comparable to those observed here.^43^ Notably, our study is the first to directly compare G8, SPPB, and a CGA-based frailty index (IF-VIG) for 1-year mortality in older cancer patients, providing clinically relevant head-to-head data.

The superior performance of SPPB and IF-VIG is clinically plausible. IF-VIG offers a comprehensive CGA-based evaluation, capturing multidomain deficits, while SPPB assesses objective physical performance, directly reflecting functional decline and imminent health deterioration. In contrast, G8, although convenient and oncology-specific, is more subjective and may underestimate risk in patients with advanced disease.

Optimized cut-off values for this cohort were lower (G8 ≤ 12.5, SPPB ≤8, IF-VIG ≥0.16) than standard thresholds. Similar G8 cut-off values emerged in 2 observational studies conducted in Taiwan and India (<13 and <12, respectively).^44^^,^^45^ These findings suggest that conventional cut-offs, developed in broader or less advanced cancer-stage populations, may under-recognize frailty in high-risk patients, and context-specific thresholds may improve prognostic accuracy and clinical decision-making. In this study, the purpose of the optimized cut-offs was to improve the clinical usability of widely adopted frailty tools by providing context-calibrated thresholds that support risk stratification, rather than to replace individualized decision-making. Importantly, this approach may be particularly useful in heterogeneous oncology cohorts, where a multidimensional, patient-centered assessment can complement disease-focused parameters and help structure care beyond tumor type alone. In practice, a context-specific classification of frailty should be viewed as a signal to trigger actions—such as identifying potentially modifiable vulnerabilities (nutrition, physical function/prehabilitation, medication review, symptom control), intensifying monitoring, and facilitating earlier goals-of-care discussions. Larger studies are needed to validate and, if necessary, refine thresholds in more specific tumor-, stage-, and treatment-defined subgroups.

Frailty, as measured by these tools, proved a stronger predictor of survival than chronological age alone, reflecting its multidimensional nature, encompassing physiological, functional, and cognitive status, whereas age alone fails to capture individual variations in health status and resilience.^46^ Sex differences in mortality were also observed, potentially reflecting biological, lifestyle, and comorbidity-related factors.^47^

From a practical perspective, SPPB offers a rapid, objective, and easily administered assessment, even by non-specialized staff, making it a valuable tool when full CGA is not feasible. IF-VIG, while highly informative, is time-intensive. G8 remains useful for initial screening but demonstrated lower prognostic accuracy. A flexible, frailty-guided approach can optimize routine oncogeriatric care by offering resource-conscious solutions tailored to diverse clinical settings while guiding individualized treatment decisions. Specifically, frailty assessment can support prognostic counselling by providing an objective, multidimensional estimate of vulnerability to inform shared decision-making, while also acting as triage triggers to activate tailored multidimensional pathways and guiding personalization of treatment delivery and follow-up intensity. Since frailty is a dynamic condition, repeated assessment may help track response to prehabilitative or supportive interventions and refine treatment discussions over time.

Strengths of this study include its prospective multicenter design, comprehensive frailty assessment, and adjustment for relevant covariates. Limitations include a modest sample size, potential selection bias due to recruitment from specific clinical settings, male predominance—likely due to the under-representation of breast and gynecological cancers—and the lack of systematic tracking of rehospitalizations during follow-up. Moreover, detailed information on prior anticancer treatments, lines of therapy, and treatment modifications, was not systematically collected. The lack of these data precluded adjustment for treatment intensity, potentially resulting in residual confounding. Future studies should prospectively capture granular treatment exposure to better contextualize the prognostic performance of frailty tools. Furthermore, future studies should examine shorter prognostic horizons and functional outcomes to complement 12-month mortality prediction.

In conclusion, all evaluated frailty tools were significantly associated with 1-year mortality in older cancer patients, with SPPB and IF-VIG outperforming G8. These findings highlight their potential utility in routine oncogeriatric practice, supporting risk stratification and personalized, goal-aligned treatment decisions, with tool selection adapted to clinical context and resource availability.

## Supplementary Material

glag099_Supplementary_Data

## Data Availability

Data are publicly available for statistical and scientific research on the CORA repository (https://dataverse.csuc.cat/dataset.xhtml?persistentId=doi:10.34810/data1965). For further inquiries, please contact the corresponding author.
