# Supplementary material for: Predicting 1-year mortality in older cancer patients: performance of G8, SPPB, and IF-VIG in the PROFIT Study
Source: J Gerontol A Biol Sci Med Sci. 2026 Apr 16;81(5):glag099. doi: 10.1093/gerona/glag099 (PMC13134773; doi:10.1093/gerona/glag099)
Supplement: glag099_Supplementary_Data [file glag099_supplementary_data.zip › 27-Apr-2026_040530_Supplemental_materials_rev_def.pdf]

Predicting One-Year Mortality in Older Cancer Patients: Performance of G8, SPPB, and IF-VIG in the PROFIT Study

Supplemental materials

eFigure 1. Calibration Plot G8

eFigure 2. Calibration Plot SPPB

eFigure 3. Calibration Plot IF-VIG

eFigure 4. Venn diagram showing overlap of patients classified as frail by G8, SPPB, and IF-VIG using optimized cut-offs

eFigure 5. Association between frailty and risk of one-year mortality, additionally adjusting for tumoral stage (sensitivity analysis, N=223)

eTable 1. Reclassification of frailty status using original versus optimized cut-offs across frailty tools

eFigure 1. Calibration Plot G8

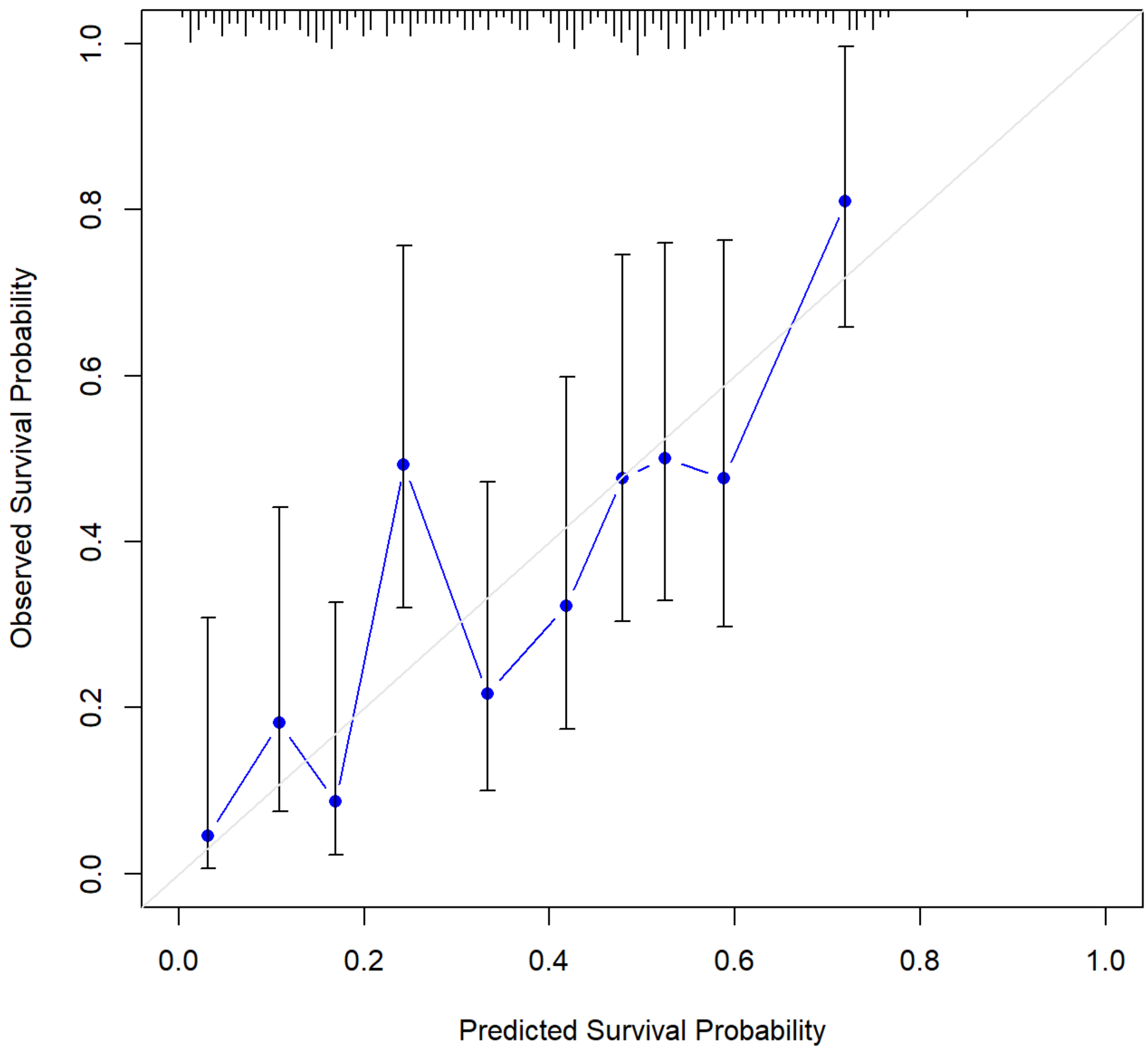

eFigure 2. Calibration Plot SPPB

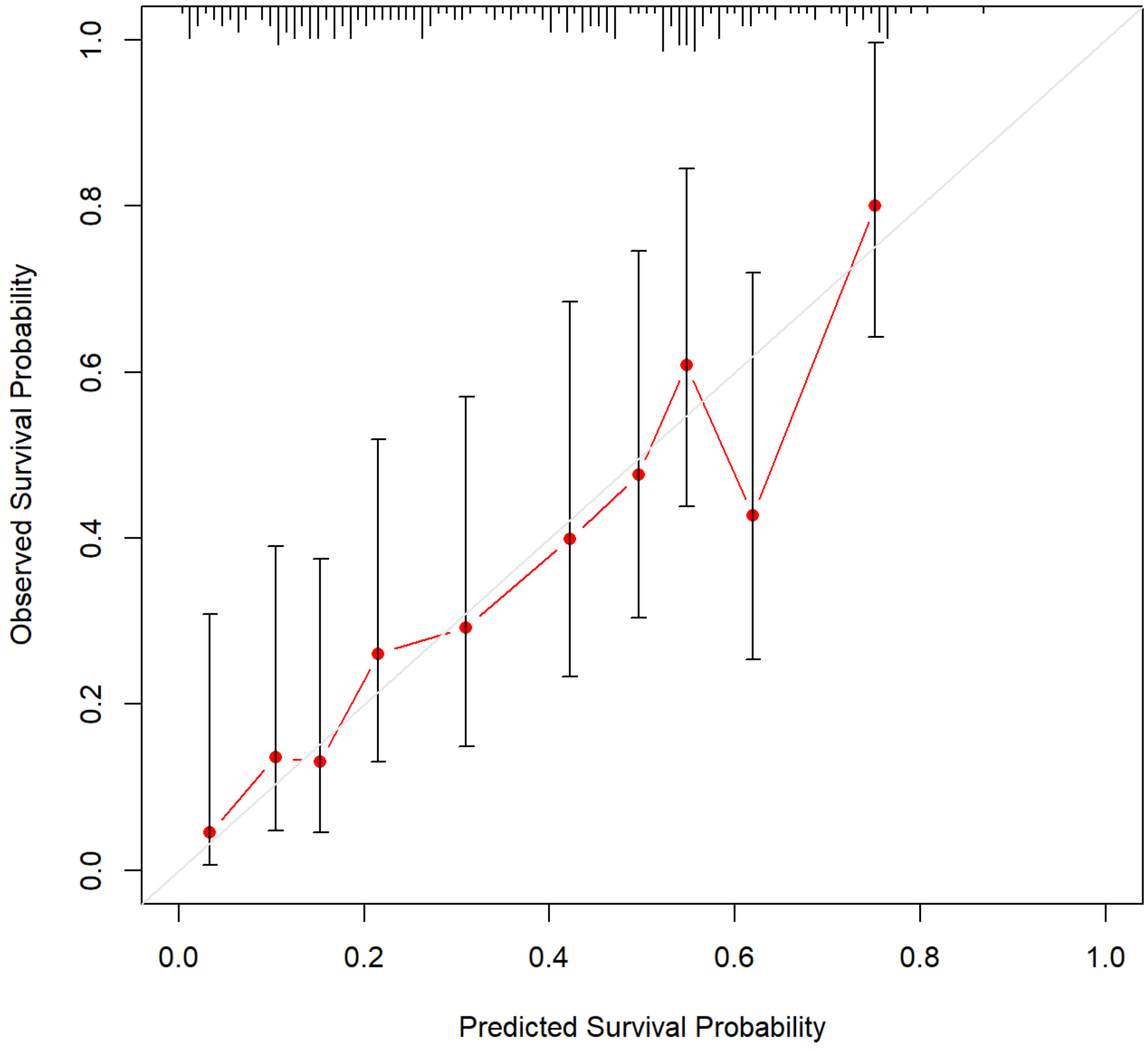

eFigure 3. Calibration Plot IF-VIG

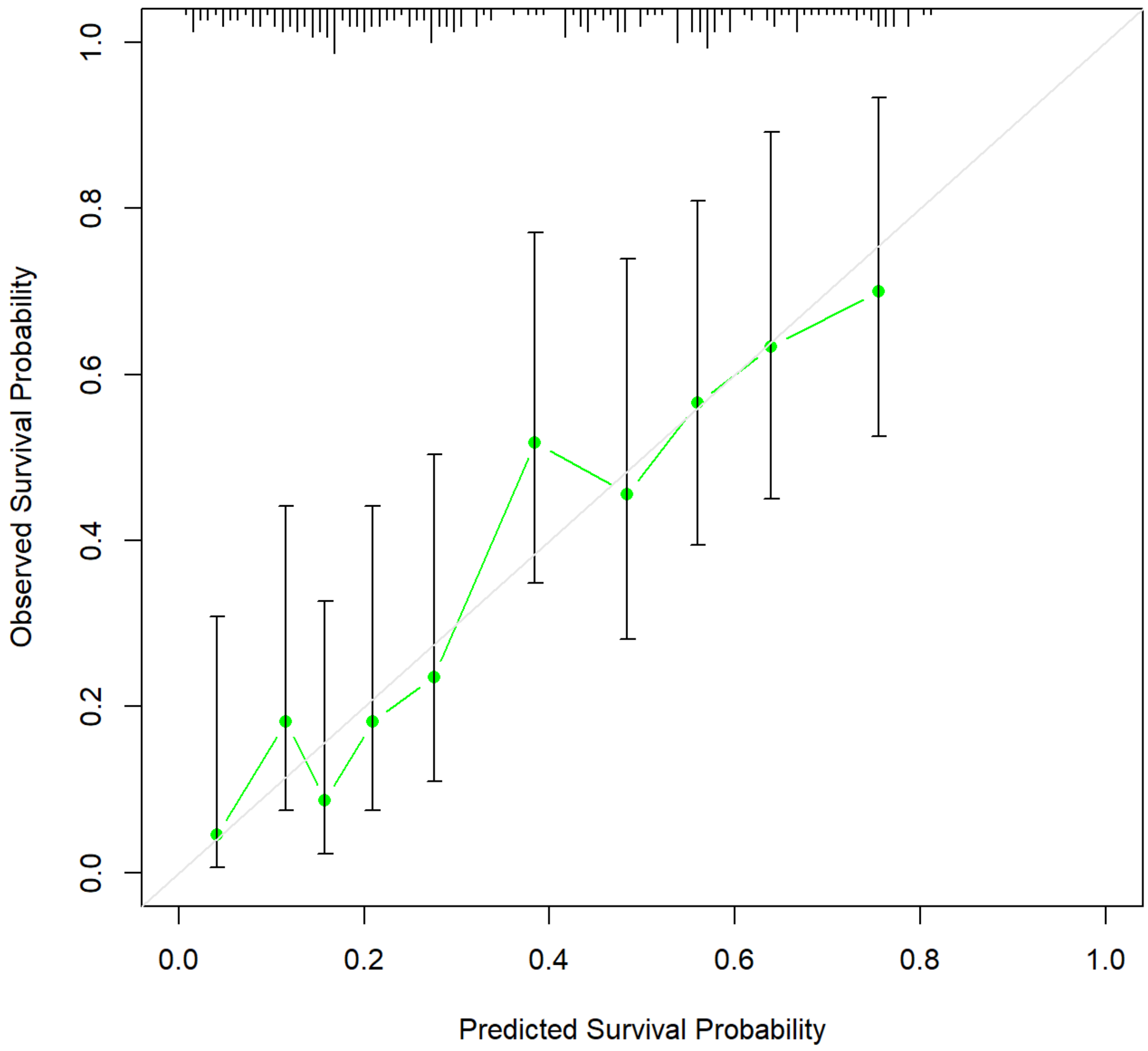

eFigure 4. Venn diagram showing overlap of patients classified as frail by G8, SPPB, and IF-VIG using optimized cut-offs

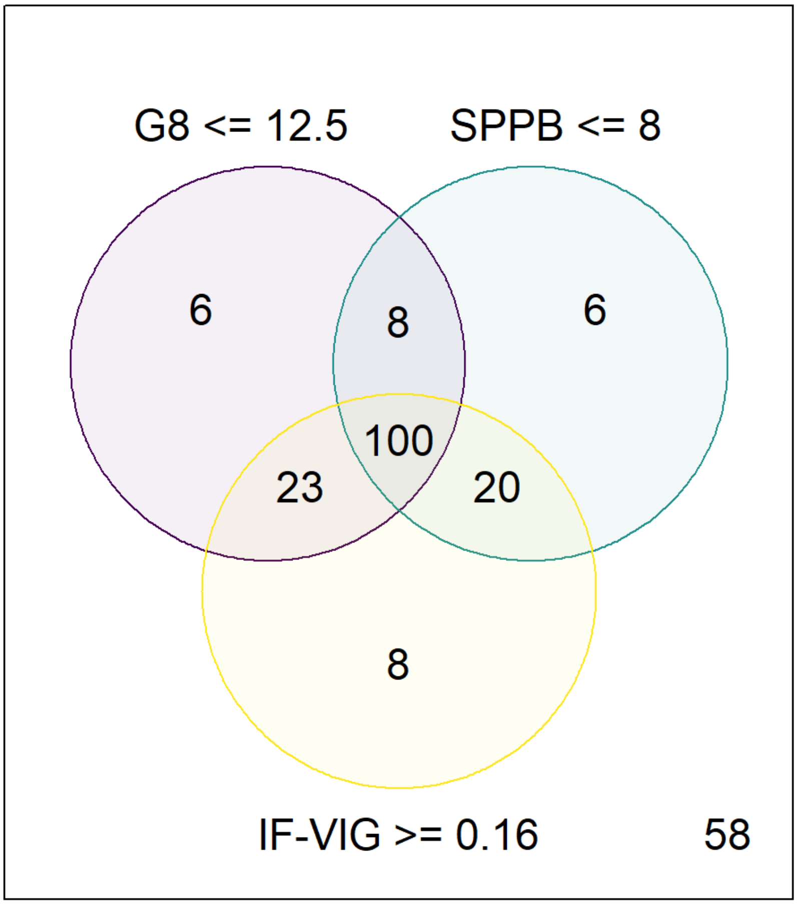

Note. G8: Geriatric 8 questionnaire; SPPB, Short Physical Performance Battery; IF-VIG Indice frágil – Valoración Integral Geriátrica

eFigure 5. Association between frailty and risk of one-year mortality, additionally adjusting for tumoral stage (sensitivity analysis, N=223)

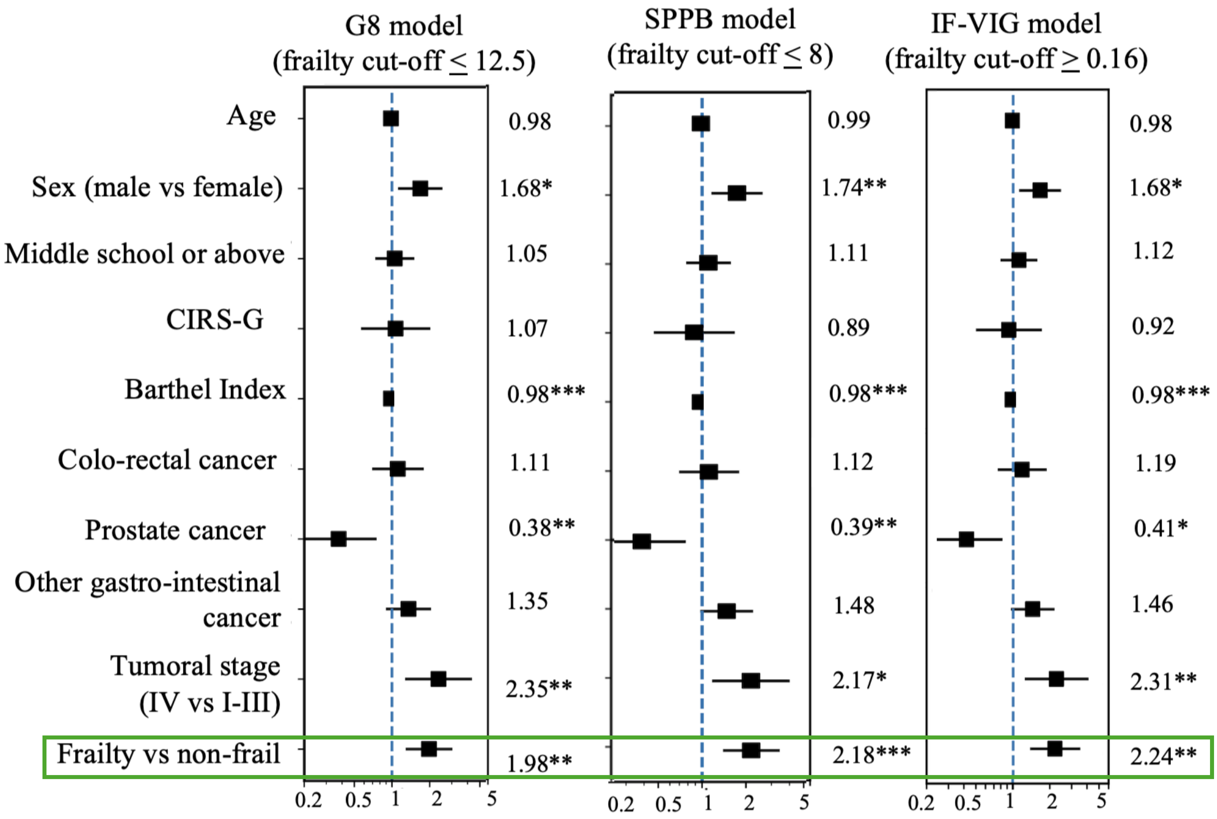

*p-value < 0.05; ** p-value < 0.01; *** p-value < 0.001

Note: Lung cancer is used as a reference for any cancer site.
CIRS-G: Cumulative Illness Rating Scale for Geriatrics; G8: Geriatric 8 questionnaire;
SPPB, Short Physical Performance Battery; IF-VIG Indice frágil – Valoración Integral Geriátrica.

eTable 1. Reclassification of frailty status using original versus optimized cut-offs across frailty tools

Frailty tool	Frail, n (%) [original cut-off]	Frail, n (%) [optimized cut-off]	Δ frail (n)	p-value*
**G8**	171 (74.7)	137 (59.8)	−34	< 0.001
**SPPB**	153 (66.8)	134 (58.5)	−19	< 0.001
**IF-VIG**	128 (55.9)	151 (65.9)	+23	< 0.001

Note. G8 (Geriatric 8 questionnaire): frail defined as ≤14 (original) vs ≤12.5 (optimized); SPPB (Short Physical Performance Battery): frail defined as ≤9 (original) vs ≤8 (optimized); IF-VIG (Indice frágil – Valoración Integral Geriátrica): frail defined as ≥0.20 (original) vs ≥0.16 (optimized).

*P-values were calculated using the exact McNemar test to compare paired classifications (frail vs non-frail) obtained with the original versus optimized cut-offs within the same participants.
