# Supplementary figures and images for: Predicting 1-year mortality in older cancer patients: performance of G8, SPPB, and IF-VIG in the PROFIT Study
Source: J Gerontol A Biol Sci Med Sci. 2026 Apr 16;81(5):glag099. doi: 10.1093/gerona/glag099 (PMC13134773; doi:10.1093/gerona/glag099)

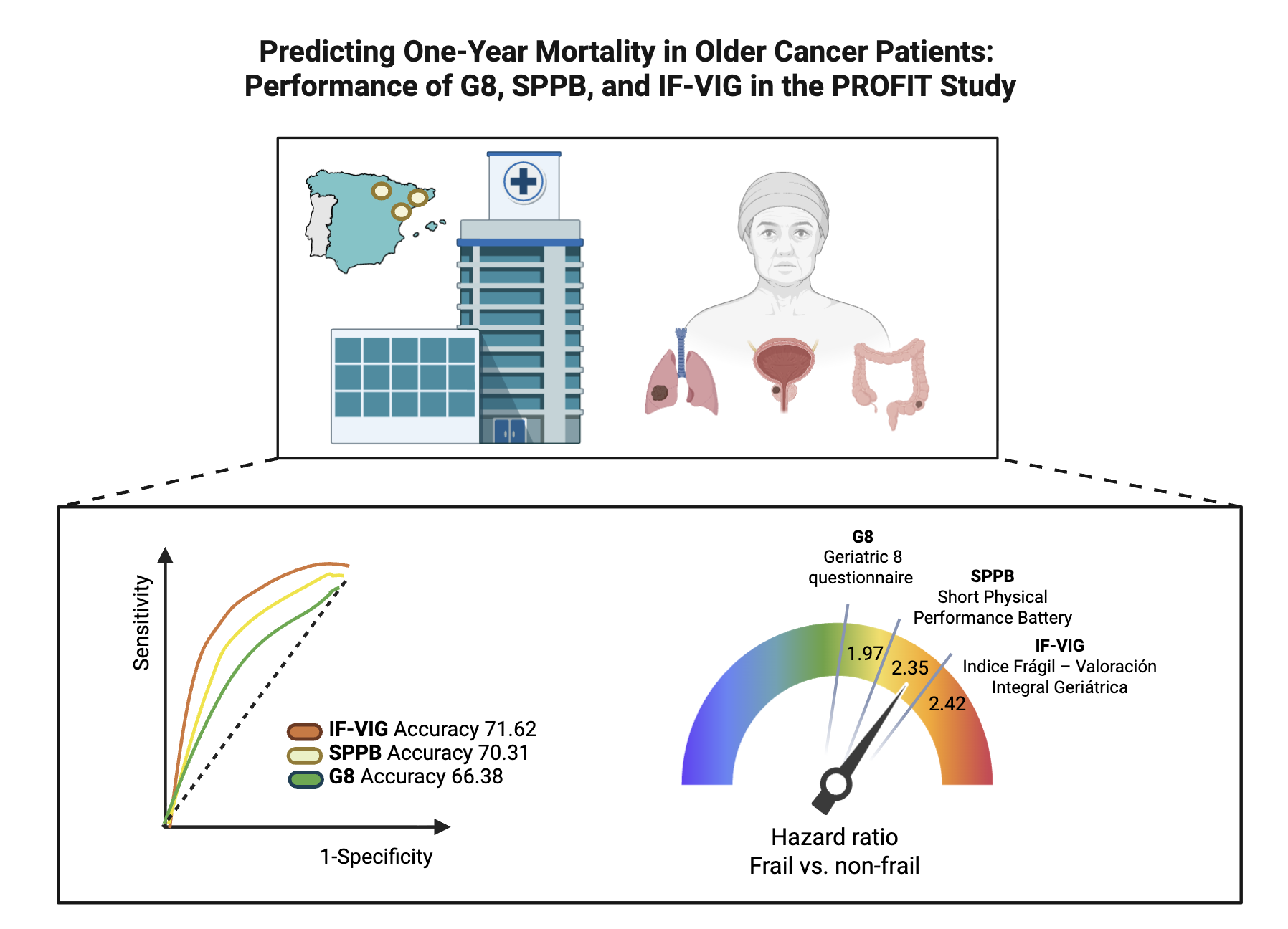

Supplement: glag099_Supplementary_Data [file glag099_supplementary_data.zip › Graphical_abstract_PROFIT.tiff]
